# Influence de l'intensité de l'intoxication tabagique sur les paramètres de sévérité des exacerbations aiguës de la broncho-pneumopathie chronique obstructive traitées en milieu hospitalier

**DOI:** 10.11604/pamj.2021.38.91.21512

**Published:** 2021-01-27

**Authors:** Ahmed Ben Saad, Ali Adhieb, Asma Migaou, Saousen Cheikh Mhamed, Nesrine Fahem, Naceur Rouatbi, Samah Joobeur

**Affiliations:** 1 Service de Pneumologie et d´Allergologie, Hôpital Universitaire Fattouma Bourguiba, Rue 1er juin, 5000 Monastir, Tunisie

**Keywords:** Broncho-pneumopathie chronique obstructive, tabagisme, exacerbation de la maladie, hospitalisation, Chronic obstructive pulmonary disease (COPD), smoking, disease exacerbation, hospitalization

## Abstract

**Introduction:**

le tabagisme constitue le principal facteur de risque de la broncho-pneumopathie chronique obstructive (BPCO). Le cours évolutif de cette maladie est caractérisé par la survenue des exacerbations aiguës (EA). L'objectif de notre travail est d'évaluer l´impact de l´intensité de l´intoxication tabagique (en paquets-années (PA)) sur les différents paramètres de sévérité des EA des patients BPCO non sevrés hospitalisés.

**Méthodes:**

c´est une étude rétrospective, monocentrique, portant sur 685 patients porteurs de BPCO, tabagiques non sevrés ayant été hospitalisés au moins une fois pour une EA entre 1990 et 2017. Nous avons défini 2 groupes de patients (G1: < 30PA, et G2: ≥ 30PA). Nous avons comparé les différents paramètres de sévérité des EA BPCO entre les deux groupes.

**Résultats:**

l´âge moyen de nos patients était de 66 ans. Il n'y avait pas de différence significative entre les deux groupes concernant l´importance du syndrome inflammatoire biologique, la durée de l´hospitalisation et celle de l´antibiothérapie. Le G2 était caractérisé par une PaO2 plus basse au cours des EA (G1: 63,5, G2: 59,3, p: 0,007), avec plus d'hospitalisation en réanimation (p < 0,001), plus de recours à la ventilation non invasive (p: < 0.001) et à la ventilation invasive (p: 0,008). Le G2 avait plus d'EA/an (G1: 2,06, G2: 2,72/patient/an, p: 0,001) avec un délai moyen de survenue d'EA sévère plus court (p: 0,038).

**Conclusion:**

l´intensité de l´intoxication tabagique a un impact négatif sur plusieurs paramètres de sévérité des EA sévères de BPCO. D´où l´intérêt de sevrage tabagique pour prévenir la maladie et ses complications.

## Abstract (en)

**Introduction:**

smoking is the leading risk factor for chronic obstructive pulmonary disease (COPD). Disease evolution is characterized by the occurrence of acute exacerbations (AE). The purpose of our study is to assess the effect of intensity of smoking intoxication (in packs-years (PAs)) on the different severity parameters of AE in smoking patients with COPD treated in hospital.

**Methods:**

we conducted a retrospective, monocentric study of 685 smoking patients with COPD who had been hospitalized at least once for an AE between 1990 and 2017. Patients were divided into 2 groups (G1: < 30PA, and G2: ≥ 30PA). The different severity parameters of COPD AE were compared between the two groups.

**Results:**

the average age of our patients was 66 years. There were no significant differences between the two groups with respect to the severity of biologic inflammatory syndrome, length of stay in hospital and antibiotic treatment duration. G2 was characterized by lower PaO2 levels during AE (G1: 63.5, G2: 59.3, p: 0.007), longer length of stay in the emergency department (p < 0,001), increased use of non-invasive ventilation (p: < 0.001) and invasive ventilation (p: 0,008). G2 had more EA/year (G1: 2.06. G2: 2.72/patient/year, p: 0.001) with a shorter mean time for severe AE (p: 0.038). **Conclusion:** the intensity of smoking intoxication has a negative impact on several severity parameters of severe COPD EAs. Hence the role of smoking cessation in preventing this disease and its complications.

## Introduction

La bronchopneumopathie chronique obstructive (BPCO) est définie comme une maladie commune qu´on peut prévenir et traiter, caractérisée par des symptômes respiratoires persistants et une limitation persistante des débits aériens dues à des anomalies des voies aériennes et/ou alvéolaires souvent causées par une exposition significative à des particules nocifs ou gaz [1]. Le diagnostic repose sur l´existence d´un trouble ventilatoire obstructif qui est défini par un rapport volume expiratoire maximum seconde (VEMS) / capacité vitale forcée (CVF) post bronchodilatation < 70% [1].

La BPCO est l´une des principaux problèmes de santé publique, non seulement en raison de sa prévalence élevée et les coûts de santé associés élevés, mais aussi en raison de la morbidité et la mortalité résultantes élevées et la diminution de la qualité de vie liée à la santé [2]. La BPCO est actuellement la quatrième cause de décès dans le monde. La projection en 2020 fait état de la BPCO comme 3^e^ cause de mortalité mondiale [3]. Le tabagisme est la principale cause de BPCO [1, 4]. En effet, environ 15 à 50% des fumeurs chroniques développent la BPCO [5, 6].

Le cours évolutif de la BPCO est caractérisé par la survenue d'exacerbations aiguës (EA) qui sont associées à une lourde morbidité et mortalité [7]. Les exacerbations de BPCO sont définies par une aggravation aiguë des symptômes respiratoires justifiant une modification thérapeutique [8, 9]. Les exacerbations aiguës, essentiellement causées par des infections bactériennes et virales, sont une cause majeure d'hospitalisation et de décès [10].

Il existe peu de données dans la littérature relatives à la relation entre l´intensité de l´intoxication tabagique et la sévérité de la BPCO, notamment la sévérité des exacerbations nécessitant l´hospitalisation. L´objectif de notre étude est d´évaluer l´impact de l´intensité de l´intoxication tabagique, en fonction du nombre de paquets années (PA), sur les différents paramètres de sévérité des EA des patients BPCO non sevrés hospitalisés.

## Méthodes

**Nature de l´étude:** il s´agit d´une étude rétrospective, portant sur les dossiers des patients BPCO tabagiques non sevrés hospitalisés pour EA au Service de Pneumologie et d´Allergologie du CHU Fattouma Bourguiba - Monastir entre janvier 1990 et décembre 2017.

**Les critères de sélection des dossiers:** les patients inclus dans cette étude sont les malades tabagiques non sevrés porteurs de BPCO selon la définition du GOLD [1]: la présence d´une symptomatologie respiratoire chronique (toux, expectorations, dyspnée d´effort), et/ou d´une histoire d´exposition aux facteurs de risque de BPCO; la présence d´un trouble ventilatoire obstructif à la spirométrie définie par un rapport VEMS/CVF < 70% post bronchodilatation. Tous les patients inclus dans cette étude sont ceux ayant eu au moins une exacerbation aiguë sévère nécessitant l´hospitalisation au service de pneumologie. Sont exclus de cette étude les patients porteurs d´affections respiratoires chroniques comportant un trouble ventilatoire obstructif permanant et ne rentrant pas dans le cadre des BPCO: l´asthme bronchique dans sa forme chronique avec dyspnée continue; le syndrome de chevauchement asthme-BPCO (asthma-COPD overlap (ACO)) selon les recommandations de GINA 2019 [11]; les bronchiolites chroniques de l´adulte; certaines formes de dilatation des bronches compliquées de trouble ventilatoire obstructif.

En tout, 685 dossiers de patients porteurs de BPCO ont été retenus pour cette étude.

**Evaluation des paramètres de sévérité des exacerbations aiguës de BPCO:** nous avons pris en considération les patients ayant été hospitalisés au moins une fois dans notre service pour EA (EA sévère). Différents paramètres de sévérité de l´exacerbation ont été évalué, comportant la gazométrie sanguine à l´admission, le syndrome inflammatoire biologique à l´admission (nombre de leucocytes, C-réactive protéine (CRP)), la durée de l´antibiothérapie, la durée de l´hospitalisation et l´évolution de l´exacerbation sous traitement (recours à la ventilation non invasive (VNI), recours à la ventilation mécanique invasive (VMI), hospitalisation en réanimation, délai de la prochaine EA sévère, nombre total des exacerbations par an). Nous avons déterminé la moyenne des différentes variables quantitatives relatives aux EA sévères pour chaque patient.

**Evaluation de l´intensité de l´intoxication tabagique:** l´intensité de l´intoxication tabagique des patients BPCO non sevrés, basé sur le nombre de paquets années (PA), a été déterminée pour chaque patient lors des consultations ou des hospitalisations.

**Impact de l´intensité de l´intoxication tabagique sur les différents paramètres de sévérité des EA de BPCO nécessitant l´hospitalisation:** afin d´évaluer l´impact de l´intensité de l´intoxication tabagique, en fonction du nombre de paquets années, sur les différents paramètres de sévérité des EA des patients BPCO non sevrés hospitalisés, nous avons défini 2 groupes de patients: Groupe 1 (G1): < 30PA; Groupe 2 (G2): = 30PA. Nous avons comparé les différents paramètres de sévérité des EA de BPCO entre les deux groupes.

**Analyse statistique:** les données ont été saisies et analysées grâce au logiciel SPSS (Version 20). Les variables quantitatives sont exprimées en moyennes ± déviations standards (DS). Les variables qualitatives sont exprimées en taux. L´analyse des variables qualitatives a été faite au moyen d´un test de Chi2. L´analyse des variables quantitatives a été faite au moyen d´un test de Student. Une valeur (p) inférieure à 0,05 était considérée comme statistiquement significative.

## Résultats

Notre étude a inclus 685 patients BPCO tabagiques non sevrés hospitalisés durant la période d'étude. La plupart des patients étaient de sexe masculin (n = 679). L´âge moyen était de 66 ± 10 ans. Le VEMS moyen était de 42 ± 15,7% de la prédite. En termes de classification GOLD, 68,3% des patients étaient classés GOLD 3 et 4. Le nombre moyen d'EA-BPCO/an était de 2,65 ± 1,72 (Tableau 1).

**Tableau 1:** caractéristiques épidémiologiques et cliniques de la population étudiée

	Nombre / Moyenne	Fréquence
**N Patients**	685	
**Age**	66 ± 10	
**Genre (M)**	679	99,1%
**Intoxication tabagique (PA)**	62 ± 26	
**Comorbiditiés ≥ 1**	586	85,6%
**VEMS (ml)**	1178 ± 489	
**VEMS (%)**	42 ± 15,7	
**VEMS / CVF (%)**	57 ± 10	
**GOLD 3 / 4**	468	68,3%
**PaO2 (mmHg)**	68,9 ± 11,8	
**PaCO2 (mmHg)**	41 ± 6,9	
**N EA / an**	2,65 ± 1,72	
**N H en Pneumologie /an**	1,18 ± 0,81	
**IRC**	378	55,2%
**OLD**	90	13,1%
**Suivi (an)**	4,2 ± 2,8	

N: nombre, M: masculin, PA: paquets-années, mMRC: modified Medical Research Council, VEMS: volume expiratoire maximum seconde, CVF: capacité vitale forcée, EA: exacerbation aiguë, H: hospitalisation, USI: unité de soins intensif, IRC: insuffisance respiratoire chronique, OLD: oxygène longue durée.

Pour étudier l´impact de l´intensité de l´intoxication tabagique sur les paramètres de sévérité des EA sévères de BPCO, nous avons comparé deux groupes de patients: G1: tabagisme < 30PA (soixante-treize patients, 10,7%); G2: tabagisme ≥ 30PA (six cents et douze patients, 89,3%).

Le G2 avait un nombre moyen des EA par an significativement plus élevé par rapport au G1 (p = 0,001). Il n´y avait pas de différence statistiquement significative entre les deux groupes en termes de pH et de PaCO2 lors des EA sévères. Par contre, le G2 avait une PaO2 et une SaO2 significativement plus basses par rapport au G1 (p = 0,007 et p=0,005 respectivement). Pour le syndrome inflammatoire biologique (taux de leucocytes et de la CRP), il n´y avait pas de différence significative entre les deux groupes. La durée moyenne de l´antibiothérapie était comparable aussi entre les deux groupes. Elle était égale à 11,3 ± 3,5 jours dans G1 et à 11,9 ± 3,6 jours dans G2 (p = 0,25). De même, la durée moyenne de l´hospitalisation était sans différence significative entre les deux groupes (p = 0,2). Le nombre d´hospitalisation en réanimation était significativement plus élevé dans G2 (p < 0,001) avec un recours plus fréquent à la VNI et à la VMI. Le délai moyen de survenue de la prochaine EA sévère de BPCO était significativement plus court dans G2 par rapport au G1 (p = 0,038) (Tableau 2).

**Tableau 2:** comparaison entre les deux groupes selon l´intensité de l´intoxication tabagique

	G1	G2	p
**pH**	7,38	7,38	0,7
**PaO2 (mmHg)**	63,5	59,3	**0,007**
**PaCO2 (mmHg)**	41,7	43,3	0,2
**SaO2(%)**	90,2	87,4	**0,005**
**GB (éléments/mm^3^)**	11598	12209	0,33
**CRP (mg/l)**	73	82	0,5
**Durée de l´antibiothérapie (jours)**	11,3	11,9	0,25
**Durée d´hospitalisation (jours)**	9	10	0,2
**Recours à la VNI (nombre/patient/an)**	0,12	0,36	**< 0,001**
**Recours à la VMI (nombre/patient/an)**	0,08	0,2	**0,008**
**Nombre des EA par an (nombre/patient/an)**	2,06	2,72	**0,001**
**Délai moyen de la prochaine EA sévère (jours)**	291	202	**0,038**
**H en réanimation (nombre/patient/an)**	0,09	0,26	**< 0,001**

GB : globule blanc, CRP : C Reactive Protein, VNI : ventilation non invasive, VMI : ventilation mécanique invasive, EA : exacerbation aiguë, H : hospitalisation.

## Discussion

Notre travail visait à étudier l´impact de l´intensité de l´intoxication tabagique, en fonction du nombre de paquets années (PA), sur les différents paramètres de sévérité des EA des patients BPCO non sevrés hospitalisés. Les patients ayant une intoxication tabagique supérieure ou égale à 30 PA avaient des désaturations plus profondes lors des hospitalisations pour EA, avec plus de recours à la VNI, à la VMI, plus d'hospitalisation en réanimation, et un délai plus court d'EA sévères. Nous avons conclu que l´intensité de l´intoxication tabagique a un impact négatif sur plusieurs paramètres de sévérité des EA sévères de BPCO. Il existe peu de données dans la littérature relatives à la relation entre l´intensité de l´intoxication tabagique et la sévérité de la BPCO, notamment la sévérité des exacerbations aiguës nécessitant l´hospitalisation. La BPCO est une maladie complexe caractérisée par une réponse inflammatoire pulmonaire anormale à la fumée de cigarette entraînant la destruction des tissus et la limitation des débits aériens. En effet, le stress oxydatif induit par la fumée de cigarette induit une inflammation chronique des poumons responsable d'une limitation irréversible des débits aériens et d´un déficit des défenses immunitaires des poumons conduisant à des infections bactériennes des voies respiratoires qui sont la principale cause d´exacerbations aiguës [12].

L'histoire naturelle de la BPCO est ponctuée par des exacerbations qui semblent accélérer le déclin de la fonction pulmonaire, entraînant une réduction de l'activité physique, une qualité de vie plus médiocre et un risque accru de décès et elles sont également responsables d'une grande proportion des coûts de soins de santé [13-16]. Elles accélèrent la progression de la maladie et sont le principal contributeur au coût économique substantiel de la BPCO, en particulier si elles nécessitent l´hospitalisation [17]. A travers notre étude nous avons montré que l´intensité de l´intoxication tabagique a un impact péjoratif sur plusieurs paramètres de sévérité des EA sévères de BPCO. Une étude grec multicentrique incluant 6125 patients porteurs d´une BPCO dont l´objectif était d´évaluer la fréquence et les facteurs de risque des EA et d´hospitalisation, n´a pas montré un impact de l´intensité de l´intoxication tabagique sur la fréquence des EA ni sur le nombre d´hospitalisation [18]. Les exacerbations de la BPCO peuvent être précipitées par plusieurs facteurs, mais les causes les plus courantes sont les infections des voies respiratoires. En effet, les EA de la BPCO sont causés principalement par des infections virales et bactériennes, alors qu´une contribution mineure est également faite par la pollution atmosphérique et d'autres facteurs environnementaux. Les infections respiratoires virales causent jusqu'à 40% à 60% de toutes les exacerbations [19].

La part du tabagisme dans la susceptibilité aux infections est démontrée dans quelques travaux. Certaines études chez l´Homme montrent que le tabagisme altère le système immunitaire et favorise les infections par le *Streptocoque pneumoniae* et le *Pseudomonas aeruginosa* [10, 12]. D´autres études expérimentales faites sur le rat montrent que le tabagisme altère le système immunitaire par différents mécanismes et augmentent le risque des infections pulmonaires bactériennes et virales [16, 20, 21]. En effet, la fumée de cigarette est un médiateur inflammatoire et qui induit une inflammation pulmonaire en endommageant la barrière épithéliale respiratoire, facilitant ainsi les infections répétées [22]. L´étude de Donovan qui a été faite sur le rat a montré que l´infection par *l´influenza virus A* et le tabagisme altèrent la sensibilité des récepteurs ß-adrénergiques des petites voies aériennes [23]. Dans notre étude, on a montré que l´intensité de l´intoxication a un impact péjoratif sur le nombre et la sévérité des EA de BPCO. Le tabagisme favorise les infections respiratoires qui sont les principales causes des EA et sachant que le meilleur prédicteur des exacerbations chez un patient est une histoire d'exacerbations [14]. Ainsi, le tabagisme engendre une perpétuation des EA. Le tabagisme qui est connu comme le principal facteur de risque de BPCO, semble être un facteur de risque des EA de BPCO, favorisant ainsi la survenue des différentes complications évolutives de la BPCO liées aux EA. D´où l´intérêt du sevrage tabagique qui reste le seul moyen pour prévenir la BPCO et ralentir le déclin de la fonction respiratoire des malades BPCO.

Bien que notre étude soit caractérisée par la taille importante de l´échantillon (N = 685), la disponibilité des variables relatives aux EA sévères et le nombre réduit d´études similaires dans la littérature, notre travail n´est pas sans limites. En effet, notre étude est rétrospective, donc on ne peut pas maitriser toutes les données cliniques et paracliniques en particulier les données bactériologiques, ce qui peut être une insuffisance dans le recueil des données et de leurs interprétations ultérieures. Nous n´avons étudié que les paramètres de sévérité des EA sévères. Ainsi, on a inclus dans cette étude que les patients BPCO hospitalisés pour EA, vue le manque de données relatives aux EA modérées. Tous nos patients étaient tabagiques non sevrés. On n´a pas apprécié l´impact du tabagisme sur les EA sévères chez les patients BPCO sevrés. D´autre part, il y avait une importante différence entre la taille de deux groupes (G1: 10,7%, G2: 89,3%). D´autres études prospectives interventionnelles prolongées prenant en compte une population large et hétérogène de patients BPCO tabagiques sevrés et non sevrés sont nécessaires afin d´évaluer l´impact du sevrage tabagique sur les paramètres de sévérité des EA de BPCO. La prise en considération des nouvelles formes de tabagisme tel que la cigarette électronique est aussi nécessaire [24, 25].

## Conclusion

Ainsi à la lumière de notre étude et la revue de la littérature, il ressort que l´intensité de l´intoxication tabagique a un impact négatif sur plusieurs paramètres de sévérité des EA sévères de BPCO. L´arrêt du tabac diminue le risque du développement de la BPCO, l´aggravation des paramètres cliniques, fonctionnels respiratoires et évolutives de la maladie et constitue le traitement essentiel de cette maladie inflammatoire bronchique. La lutte anti-tabac est un enjeu important de santé publique nécessitant la participation de nombreux intervenants afin d´appliquer la convention cadre de lutte antitabac (CCLAT) de l´OMS.

### Etat des connaissances actuelles sur le sujet

- Le principal facteur de risque de la BPCO est le tabagisme;
- La relation entre l'importance de l'intoxication tabagique et la sévérité du déclin de la fonction respiratoire est connue.

### Contribution de notre étude à la connaissance

- Il existe très peu d'études qui précisent la relation entre l'importance de l'intoxication tabagique et la sévérité des exacerbations aiguës de BPCO;
- Notre travail montre que l'intoxication tabagique agit sur un facteur pronostique important au cours de la BPCO qui est l'exacerbation aiguë.
